# Genetic and Functional Studies of the Intervertebral Disc: A Novel *Murine* Intervertebral Disc Model

**DOI:** 10.1371/journal.pone.0112454

**Published:** 2014-12-04

**Authors:** Dominic W. Pelle, Jacqueline D. Peacock, Courtney L. Schmidt, Kevin Kampfschulte, Donald J. Scholten, Scott S. Russo, Kenneth J. Easton, Matthew R. Steensma

**Affiliations:** 1 Laboratory of Musculoskeletal Oncology, Center for Skeletal Disease and Tumor Metastasis, Van Andel Institute, Grand Rapids, Michigan, United States of America; 2 Department of Orthopaedic Surgery, Grand Rapids Medical Education Partners, Grand Rapids, Michigan, United States of America; 3 Department of Surgery, Michigan State University College of Human Medicine, Grand Rapids, Michigan, United States of America; 4 Section of Spine Surgery, Orthopaedic Associates of Michigan, Grand Rapids, Michigan, United States of America; 5 Department of Surgery, Spectrum Health Medical Group/Helen DeVos Children's Hospital, Grand Rapids, Michigan, United States of America; University of Michigan, United States of America

## Abstract

Intervertebral disc (IVD) homeostasis is mediated through a combination of micro-environmental and biomechanical factors, all of which are subject to genetic influences. The aim of this study is to develop and characterize a genetically tractable, *ex vivo* organ culture model that can be used to further elucidate mechanisms of intervertebral disc disease. Specifically, we demonstrate that IVD disc explants (1) maintain their native phenotype in prolonged culture, (2) are responsive to exogenous stimuli, and (3) that relevant homeostatic regulatory mechanisms can be modulated through *ex-vivo* genetic recombination. We present a novel technique for isolation of murine IVD explants with demonstration of explant viability (CMFDA/propidium iodide staining), disc anatomy (H&E), maintenance of extracellular matrix (ECM) (Alcian Blue staining), and native expression profile (qRT-PCR) as well as *ex vivo* genetic recombination (*mT/mG* reporter mice; AdCre) following 14 days of culture in DMEM media containing 10% fetal bovine serum, 1% L-glutamine, and 1% penicillin/streptomycin. IVD explants maintained their micro-anatomic integrity, ECM proteoglycan content, viability, and gene expression profile consistent with a homeostatic drive in culture. Treatment of genetically engineered explants with cre-expressing adenovirus efficaciously induced *ex vivo* genetic recombination in a variety of genetically engineered mouse models. Exogenous administration of IL-1ß and TGF-ß3 resulted in predicted catabolic and anabolic responses, respectively. Genetic recombination of *TGFBR1^fl/fl^* explants resulted in constitutively active TGF-ß signaling that matched that of exogenously administered TGF-ß3. Our results illustrate the utility of the *murine* intervertebral disc explant to investigate mechanisms of intervertebral disc degeneration.

## Introduction

Low back pain (LBP) is a significant healthcare concern worldwide [1] and is a leading reason for seeking medical attention with the lifetime prevalence approaching 84% [2]. Degeneration of the intervertebral disc (IVD) is a major contributing factor to LBP. The presence of individuals over the age of 50 with evidence of IVD degeneration (IVDD) approaches 90%, correlating with the lifetime prevalence of LBP [3].

The IVD is the cartilaginous joint between two vertebral bodies. It is comprised of an outer ring of collagen lamellae, the annulus fibrosus (AF), an inner gelatinous nucleus pulposus (NP), and two cartilaginous end plates (CEP) lying adjacent to the superior and inferior vertebral bodies. The AF is comprised primarily of type I collagen, while the NP is comprised of type II collagen and proteoglycans, principally aggrecan [4].

Due to the complex structure and distinct characteristics of the IVD tissues, challenges in translating *in vitro* assays to overall organ function persist. Monolayer culture expansion of both AF and NP tissue results in down regulation of collagen and proteoglycan production compared to freshly isolated tissue. However, phenotypic genetic expression can be restored with sophisticated micro-mass culture techniques [5], [6]. While this has been instrumental in deciphering the specific cellular characteristics of a particular IVD tissue, mechanisms of IVD organ homeostasis remains elusive. Certainly, dysfunction of a particular IVD constituent will affect the function of another under *in vivo* conditions [7], [8].

To assess IVD organ function, *ex vivo* whole IVD organ culture models have been developed. These explant models provide a useful tool to study the IVD. Insofar, rabbit, rat, sheep, bovine, and human explants have been investigated [9], [10], [11], [12], [13], [14], [15], [16], [17]. Functional assays utilizing these models have provided insight into cellular response and resultant anatomic changes in conditions simulating degeneration *in vivo* [18] and have shed light on the etiology of IVDD [19]. While these models are useful constructs to study the IVD, inherent difficulty lies in deciphering genetic mechanisms of IVD degeneration. Classic twin studies have estimated the heritability index of IVDD to be 74% in the lumbar spine [20]. Indeed, over 20 genetic polymorphisms have been identified in association with IVDD, reviewed in [21]. These data underscore the need to account for both genetic and micro-environmental factors when studying the IVD. Therefore, in comparison to other IVD organ culture models, an advantageous novel IVD organ culture model would require the potential for genetic manipulation and ability to evaluate functional organ response. Herein, we have developed a genetically tractable *murine* IVD explant culture. What follows is a characterization of the *murine* IVD as an organ culture model, verification of *ex vivo* genetic tractability, and examples of utility in functional assays. We also provide a demonstration of coupling genetic engineered models with functional IVD organ response, *ex vivo*.

## Methods

### Mice

Van Andel Institute Institutional Animal Care and Use Committee approved this study. Experiments were carried out according to institutional animal care and use committee protocols. Wild-type mice from c57bl/6, FVB, and mixed background were utilized. For experiments utilizing transgenic mice, *mT/mG*, *TGFßR1^fl/fl^*, and *NF1^fl/fl^* were used. *mT/mG* reporter mice fluoresce red at baseline; however, when exposed to cre-recombinase, they express green fluorescence protein and are useful to visually track cellular genetic recombination events [22]. *TGFßR1^fl/fl^* mice constitutively activate *TGFß* receptor 1 when exposed to cre-recombinase [23] while *NF1^fl/fl^* mice under the same conditions exhibit loss of neurofibromatosis1 function [24] and have demonstrable effects on the IVD [25].

### Explant harvest

**Figure 1:**
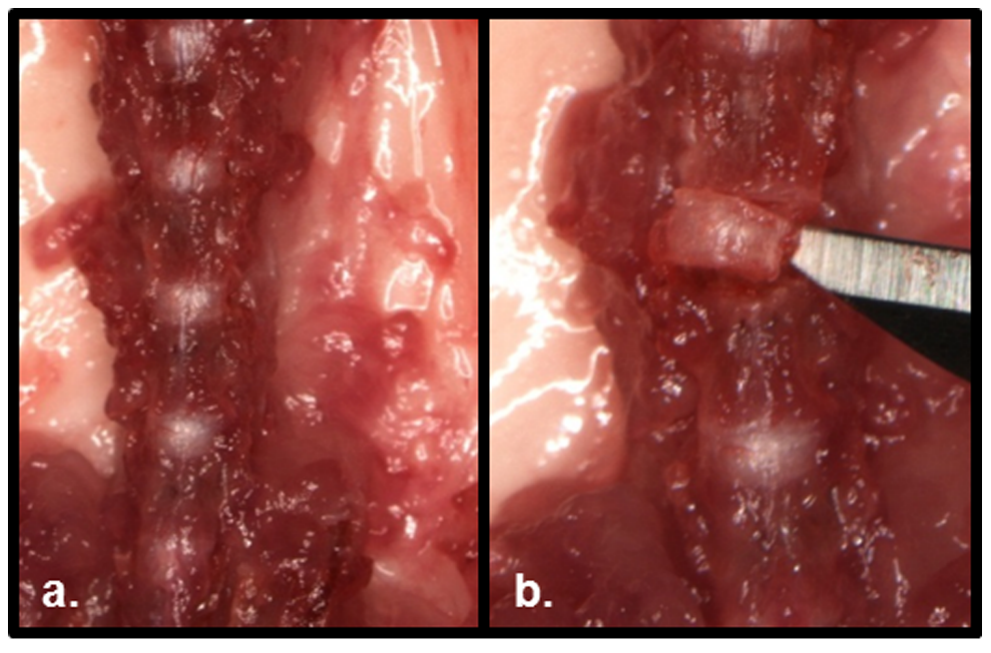
Method of IVD organ harvest for ex vivo culture. (**a**) Anterior spine is exposed after removal of peritoneal sac and paraspinal musculature. (**b**) Excision of a lumbar IVD after the superior and inferior extents were freed with sharp dissection at 4× magnification.

Mice were euthanized with carbon dioxide asphyxiation and washed with ethanol. The skin was dissected free from the anterior abdomen and thorax and subsequently discarded. The peritoneal sac was removed *en bloc*. The contents of the thoracic cavity were removed and the ribs transected transversely at their midpoint for increased exposure. With the aid of a dissecting scope (Leica) the prevertebral tissue and psoas muscles were removed, exposing the anterior spine (figure 1a). The anterior longitudinal ligament was removed with dissecting scissors and an 11 blade scalpel. The junction of the bony endplate (BEP) and CEP was identified. An 11 blade scalpel was used to transect the superior CEP from the BEP (figure 1b). The inferior vertebral body was transected through cortical bone in the upper third region. The IVD and remaining inferior vertebral body was removed from the spine. A scalpel was used to remove the remaining bony tissue from the IVD. The IVD was inspected with the dissection microscope for gross anatomic integrity. Slight anterior to posterior compression was applied with forceps; if any extravasation of NP tissue occurred during compression, the IVD was discarded. IVDs that passed compressive inspections were immediately placed in ice cold PBS. Individual discs were then placed in Dulbecco's Modified Eagle Medium containing 10% fetal bovine serum, 1% L-glutamine, and 1% penicillin/streptomycin. Media was changed every other day; explants were cultured for up to 14 days.

### Histology

For histological analysis, explant IVDs were fixed with 10% neutral buffered formalin for 24 hours, embedded in paraffin blocks, and 5 µm sections were prepared. Slides were de-paraffinized in Citrisolv (Fisher; Pittsburgh, PA), rehydrated through ethanol series and stained with hematoxylin and eosin or alcian blue.

### Cell viability

Explant IVDs were incubated with CellTracker Green CMFDA (5-Chloromethylfluorescein Diacetate) (Invitrogen) and propidium iodide (Sigma-Aldrich) according to manufacturer's protocol. Explants were rotated for 10 minutes at 4 degrees Celsius. Explant IVDs were then incubated at 37 degrees Celsius and 5% CO_2_ for 1 hour. 1/2 of explants were incubated in the presence of 70% methanol, serving as a positive control for cellular death. After fixing overnight at 4 degrees Celsius in 10% neutral buffered formalin, explants were dehydrated by immersion in 15% and 30% sucrose solutions for 24 hours at 4 degrees Celsius. Explants were imbedded in O.C.T. compound (Tissue-Tek) and frozen over dry ice. IVDs were kept at −80 degrees Celsius until sectioning. Sections were cut in 5 µm sections and imaged on a digital inverted fluorescent microscope (EVOSfl, fisher scientific).

### Allele-specific PCR

Genomic DNA was harvested from tissues using DirectPCR reagent (Viagen) according to the manufacturer's instructions. PCR reactions were performed with Syzygy MeanGreen Taq Master Mix (Syzygy Biotech) in 20 µl reactions with 1 µl crude DNA lysate. Primers designed to detect the mutated *NF1^flx^* allele (flanked by *loxp* sites, 350 base pair amplicon) and cre-recombined *NF1* allele (280 base pair amplicon) and cycling conditions were used exactly as previously described [24].

### Quantitative real-time PCR

Total RNA was extracted from explant IVDs using TRIzol reagent (Invitrogen). Synthesis of cDNA was performed using 1 µg of RNA according to the High Capacity cDNA Reverse Transcription Kit (Invitrogen). Primers (table 1) were obtained from Integrated DNA technologies. Quantitative real-time PCR reactions were performed using SYBR, Select Mastermix (Applied Biosystems) in 10 ul reactions. PCR was performed according to manufacturer's instructions using a StepOnePlus cycler (Applied Biosystems; Carlsbad, CA). Relative gene expression was normalized to GAPDH, measured by quantitative real-time PCR, and expressed using the ΔΔCT method. The paired Student's t test was utilized to identify statistically significant differences in gene expression; significance was set to a *p* value <0.05.

**Table 1:** *Murine* primers for genes analyzed.

Primer	Forward Sequence	Reverse Sequence
GAPDH	5′-AACTTTGGCATTGTGGAAGG-3′	5′-GGATGCAGGGATGATGTTCT-3′
ACAN	5′-ACCCGGTACCCTACAGAGAC-3′	5′-GTCCACCCCTCCTCACATTG-3′
ADAMTS4	5′-GAGTCCCATTTCCCGCAGAA-3′	5′-ATAACCGTCAGCAGGTAGCG-3′
COL1	5′-CTGACGCATGGCCAAGAAGA-3′	5′-TCTCACCATTGGGGACCCTT-3′
COL2	5′-CATCTTGCCGCATCTGTGTG-3′	5′-TGCCCCTTTGGCCCTAATTT-3′
TIMP1	5′-GATACCATGATGGCCCCCTTT-3′	5′-CGCTGGTATAAGGTGGTCTCG-3′
TIMP2	5′-CAGGTACCAGATGGGCTGTG-3′	5′-TGGTGCCCATTGATGCTCTT-3′

### Explant treatments

Explants were harvested and cultured in accordance with the aforementioned conditions. After 48 hours of culture, explants were treated with IL-1ß at 10 ng/mL for 12 or 24 hours to simulate degenerative conditions. For cultures simulating anabolic conditions, TGF-ß3 at 10 ng/mL for 24 hours was utilized.

For genetic recombination experiments in *mT/mG* [22], *NF1^fl/fl^* mice, and *TGFßR1^fl/fl^* explants were harvested. Following 24 hours of serum and antibiotic free culture, explants were treated with either cre-expressing adenovirus (5×10^7^ pfu/mL) or a non-cre-expressing adenovirus control (5×10^7^ pfu/mL). Serum was replaced to the culture conditions after 24 hours. At 48 hours, the explants were washed and cultured according to the aforementioned protocol. Explants were harvested from culture at 10 days and used for experimentation.

### Western blot analysis

Explants were then washed in ice cold PBS and sharply morsilized with a 15 blade scalpel in the presence of lysis buffer (RIPA lysis buffer) supplemented with complete Protease Inhibitor Cocktail (Roche). Protein was measured using the BCA assay (Pierce), and 20 µg of explant lysate was run on a 10% SDS/polyacrylamide gel. The proteins were transferred onto a nitrocellulose membrane, and membranes were probed overnight at 4 degrees Celsius with primary antibody. P44/P42(MAPK) 1∶500 (cell signaling), p-P44/P42 (activated MAPK) 1∶500 (Cell Signaling), primary antibodies were utilized. Membranes were then probed with horseradish-peroxidase-conjugated secondary antibody for 1.5 hours at room temperature before detection using an enhanced chemiluminescence (ECL) detection system (Pierce).

### Confocal microscopy

Explants of *mT/mG* mice were treated with cre-expressing adenovirus or non-cre-expressing adenovirus, as described above. Following culture for 10 days, explants were then fixed overnight at 4 degrees Celsius in 10% neutral buffered formalin, dehydrated by immersion in 15% and 30% sucrose solutions for 24 hours at 4 degrees Celsius, imbedded in O.C.T. compound (Tissue-Tek) and frozen over dry ice. IVDs were kept at −80 degrees Celsius until sectioning. Sections were cut in 5 µm sections. Slides were dried and mounted in Vectashield (Vector Labs). All images were obtained using a 20× objective on an A1plus confocal system (Nikon) with GFP and Tomato fluorescence imaged in series. Maximum intensity projection-based Z-stack image rendering was performed in NIS-Elements software (Nikon).

## Results

### Explants retain micro-anatomic integrity and cellular viability in *ex vivo* culture

**Figure 2:**
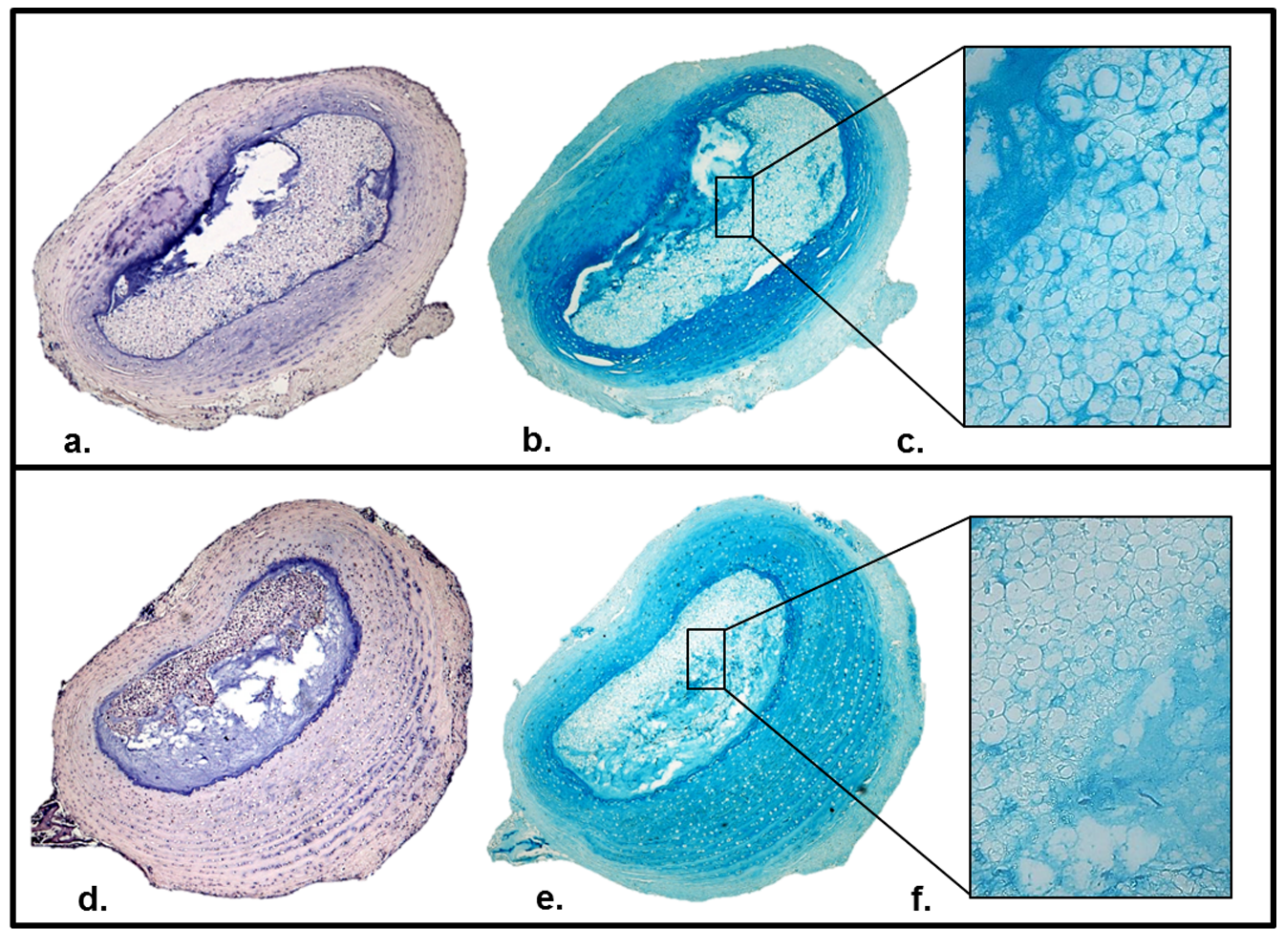
Histological analysis of explant micro-anatomic features. IVDs are shown in axial slices. (**top pane**) Freshly harvested murine IVDs. (**bottom pane**) Murine IVDs after 14 days of *ex vivo* culture. (**a,d**) Hematoxylin and eosin (**b,e**) Alcian blue. (**c,f**) 40× magnification of alcian blue stained NP.

Explants were harvested and cultured for 14 days. Histological comparison of freshly harvested and cultured explants reveals a retained integrity of micro-anatomic features (figure 2). The AF and NP were clearly demarcated. The collagen lamellar bundles of the AF were preserved in culture. The NP stained positively for proteoglycan. After 14 days of culture, the cellular architecture of the NP demonstrated characteristic vacuolated cells when examined under 40× magnification.

**Figure 3:**
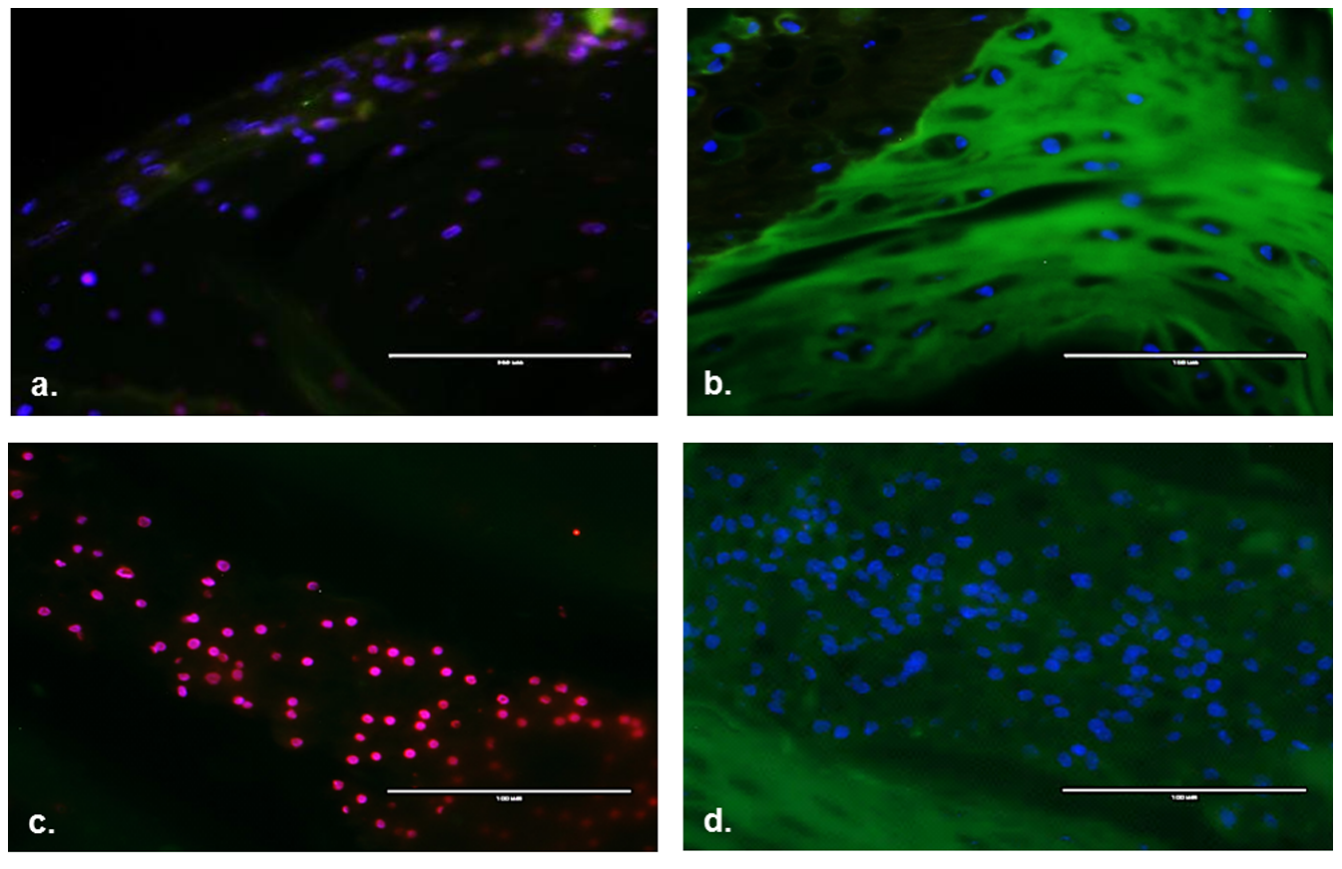
Cell viability in 14 day explant organ culture. Sections were stained with propidium iodide (red), Cell tracker green (green), and DAPI nuclear co-stain. Annulus fibrosus tissue section after treatment with 70% methanol (positive control) for 24 hours (**a**) and after 14 days of *ex vivo* organ culture (**b**). Nucleus pulposus tissue section after treatment with 70% methanol for 24 hours (**c**) and after 14 days of ex-vivo organ culture (**d**). Purple staining is indicative of PI and DAPI counterstaining.

Explants incubated with CMFDA, a cell permeable chemical that live cells will convert to a cell impermeable green fluorescent compound [26], and PI, a DNA binding red fluorescent dye impermeable to living cells [27], were imaged on a digital inverted fluorescent microscope. IVDs treated with 70% methanol demonstrated strong PI uptake and no CMFDA signal, indicative of widespread cellular death (figure 3). Explants incubated at 14 days did not demonstrate appreciable PI uptake in either the NP or AF. CMFDA uptake was noted in both the AF and NP of cultured explants.

### Explants demonstrate characteristic gene expression patterns in *ex vivo* culture

**Figure 4:**
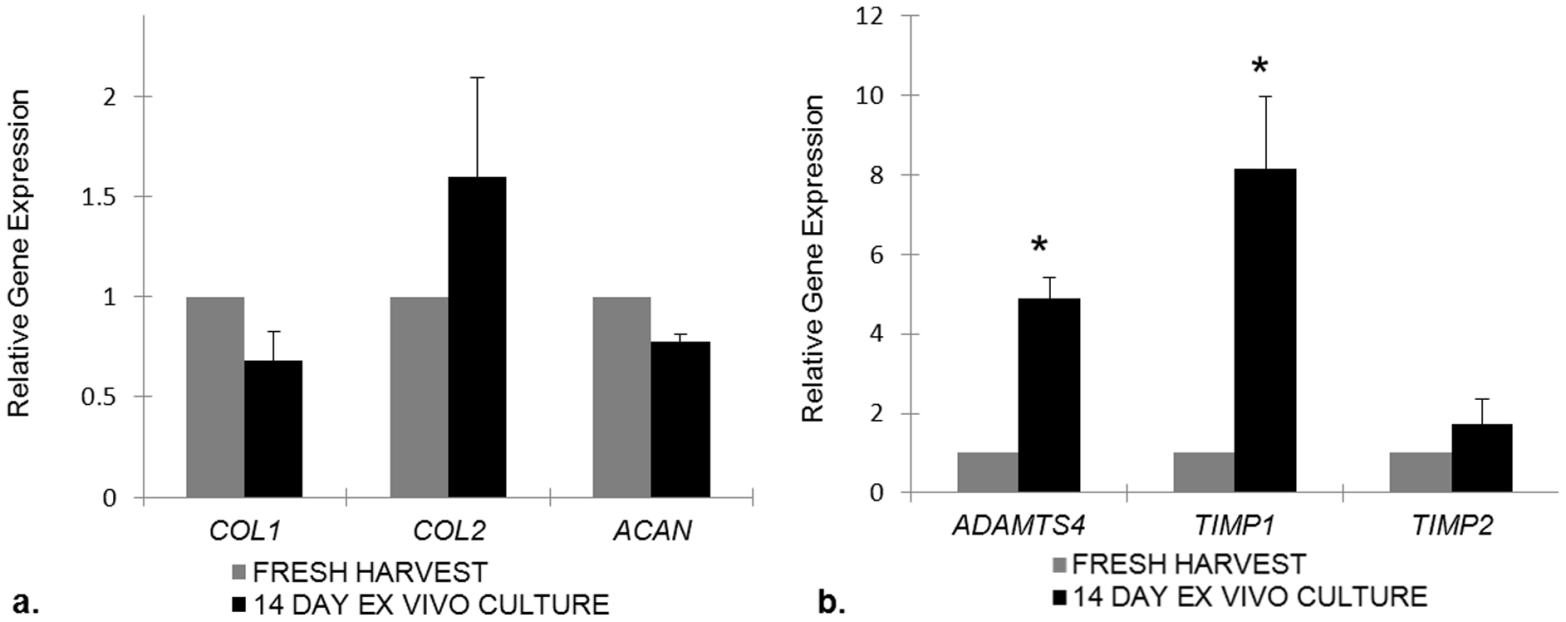
Real-time PCR analysis of 14 day murine IVD explants compared to freshly harvested IVD explants. Genes related to structural extracellular matrix production (**a**) and extracellular matrix remodeling genes (**b**) were analyzed. After 14 days in culture, murine IVD explants do not change the gene expression of the principal structural genes of the IVD, while ADAMTS4 and TIMP1 are significantly upregulated. Analysis was normalized to GAPDH. Results are presented relative to freshly harvested murine IVDs where the expression value was set to 1. * denotes statistical significance (p<0.05).

We chose to compare gene expression of *COL1*, the principal extracellular matrix protein of the AF, as well as *ACAN* and *COL2*, the principal extracellular matrix proteins of the NP, between 14 d explants and freshly harvested IVDs. No statistically significant difference in gene expression was identified in *COL1* (fold change: 0.7; 0.5–1.0; *p* = 0.3), *COL2* (fold change: 1.6; 0.7–2.3; *p* = 0.5), and *ACAN* (fold change: 0.8; 0.7–0.8; *p* = 0.7) (figure 4a). Next, we assessed the gene expression of the protease *ADAMTS4* and the protease inhibitors: *TIMP1* and *TIMP2* (figure 4b). We found a statistically significant increase in both the tissue protease *ADAMTS4* (fold change: 4.9; 3.9–5.2; *p* = 0.007) and the protease inhibitor *TIMP1* (fold change: 8.1; 5.7–11.8; *p* = 0.01), indicating that homeostatic mechanisms remain intact at day 14 in the cultured explants.

### IVD genetic recombination is successful in *ex vivo* culture conditions

**Figure 5:**
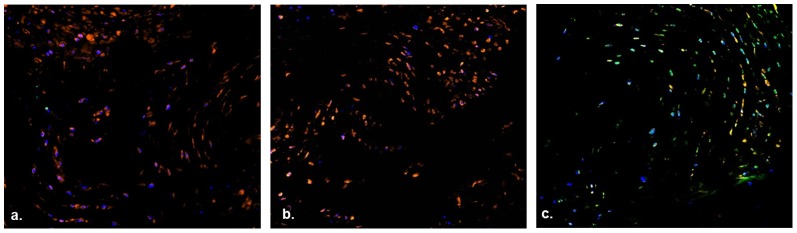
Demonstration of ex vivo murine IVD genetic recombination. *mT/mG* mice IVDs were cultured for 10 days in the presence of (**a**) standard culture conditions, (**b**) control adenovirus, or (**c**) cre-recombinase expressing adenovirus. Images are 20× magnification Slides are counterstained with DAPI (blue). Red fluorescence represents no genetic recombination, green fluorescence represents successful genetic recombination, and yellow fluorescence represents an ongoing genetic recombination event.

Wild type and *mT/mG* reporter explants were treated with cre-expressing adenovirus and control adenovirus. Both untreated and adenovirus controls failed to demonstrate GFP fluorescence conversion, while explants treated with cre-expressing adenovirus demonstrated evidence of genetic recombination *ex vivo* with GFP signal demonstrated in both the AF and NP (figure 5).

To further assess the ability of the *murine* explants to undergo genetic *ex vivo* recombination, *NF1^fl/fl^* IVD explants were cultured with cre-expressing adenovirus. Individual IVDs were subjected to allele specific PCR after culture. All individual IVDs cultured with cre-expressing adenovirus demonstrated evidence of genetic recombination (figure S1).

### Explant Response to IL-1ß

**Figure 6:**
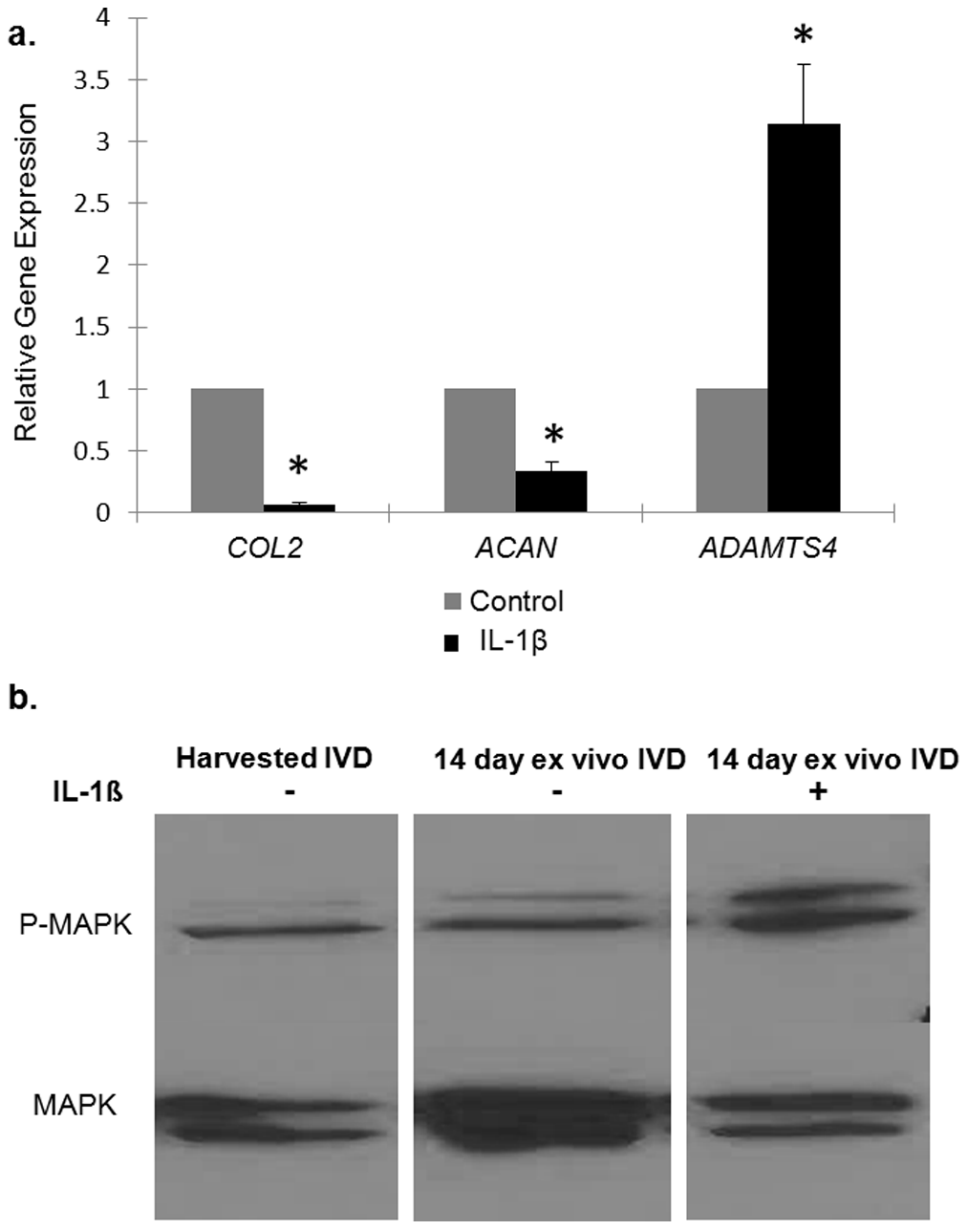
IVD explant response to IL-1ß. (**a**) When treated with IL-1ß at 10 ng/mL for 12 hours, IVD explants demonstrate significant decreases in gene expression of *COL2* (*p = 7.4×10^−11^*) and *ACAN* (*p = 3.0×10^−5^*); ADAMTS4 (*p* = 0.009) gene expression is increased. Values are normalized to *GAPDH*. Results are presented relative to IVDs cultured without IL-1ß where the expression value was set to 1 (**b**) Murine IVD explants demonstrate increased MAPK phosphorylation when treated with IL-1ß at 10 ng/mL for 24 hours. * denotes statistical significance (p<0.05).

IL-1ß is a pro-catabolic factor in IVDD [28], [29] and has been shown to stimulate IVDD both *in vitro* and *in vivo* experiments [30], [31], [32]. Upon challenge with IL-1ß (10 ng/ml), wild-type IVD explants demonstrate a significant down regulation of *COL2* (fold change: 0.07; 0.05–0.08; *p = 7.4×10^−11^*) and *ACAN* (fold change: 0.3; 0.2–0.5; *p = 3.0×10^−5^*) while *ADAMTS4* (fold change: 3.1; 2.5–4.2; *p* = 0.009) gene expression was upregulated (figure 6a). Exogenous IL-1ß administration induced sustained MAPK phosphorylation at 14 days (figure 6b) [33].

### Explant Response to TGF-ß

**Figure 7:**
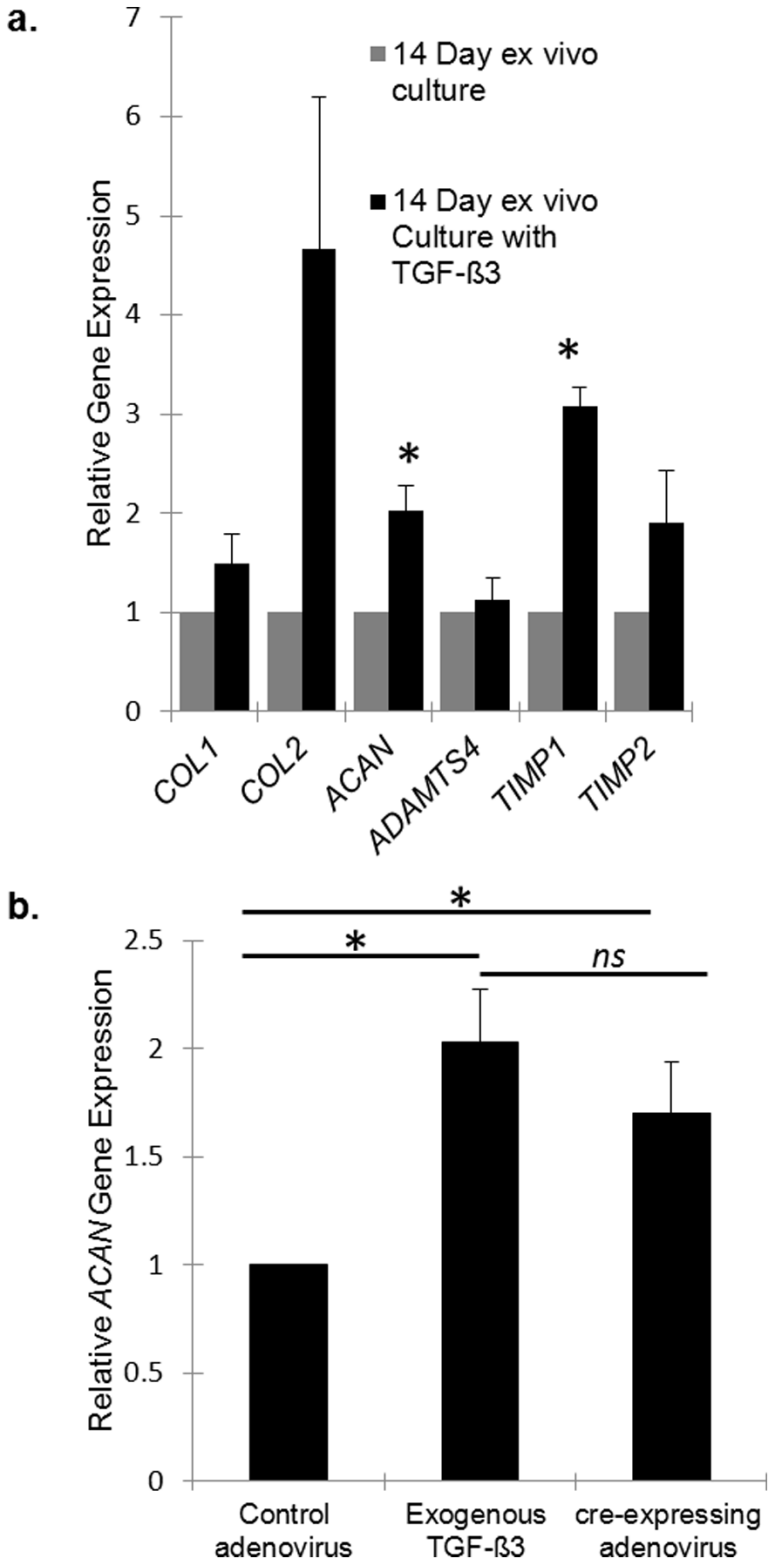
IVD explant response to TGF-ß. (**a**) Real-time PCR analysis of murine IVD explant gene expression response to stimulation with exogenously administered TGF-ß3 (10 ng/mL for 24 hours) demonstrates significant increase in the gene expressions of *ACAN* and *TIMP1*. Values are normalized to *GAPDH*. Results are presented relative to IVDs cultured without TGF-ß3 where the expression value was set to 1. Values are normalized to *GAPDH*. (**b**) Murine IVDs from *TGFßR1^fl/fl^* demonstrate significant upregulation in *ACAN* gene expression when cultured with cre-expressing adenovirus. Exogenous TGF-ß3 at 10 ng/mL for 24 hours was cultured with wild-type murine IVDs. Results are presented relative to IVDs cultured with control adenovirus where the expression value was set to 1. Values are normalized to *GAPDH*. * denotes statistical significance (*p*<0.05). *ns* denotes not statistically significant (*p*>0.05).

We chose to investigate TGF-ß for its stimulatory and protective effects on the IVD [34], [35], [36], [37]. Explants treated with exogenous TGF-ß3 (10 ng/mL) demonstrated statistically significant gene expression upregulation of *ACAN* (fold change: 2.0; 1.8–2.5; *p* = 0.005) and *TIMP1* (fold change: 3.1; 2.7–3.4; *p* = 0.007), implicating an anabolic and protective effect within the cultured IVD explant (figure 7a). *COL2* gene expression demonstrated a more variable response to TGF-ß3 but trended towards upregulation (fold change 4.7; 2.1–7.4; *p* = 0.06).

To evaluate whether IVD explants could be used to couple genetic recombination into a functional response, *TGF-ßR1^fl/fl^* mice were utilized. Tissues in *TGF-ßR1^fl/fl^* mice demonstrate constitutive activation of the TGF-ß1 receptor following recombination [23]. Aggrecan gene expression was significantly upregulated in treated *TGF-ßR1^fl/fl^* explants (fold change: 1.7; 1.0–2.2; *p* = 0.01) compared to controls (figure 7b). No difference in *ACAN* expression was observed between explants treated with exogenous TGF-ß3 and the recombined *TGF-ßR1^fl/fl^* explants (*p* = 0.4).

## Discussion

Herein, we describe a novel technique for utilizing *murine* IVD explants as a functional unit to study IVD homeostasis and degeneration. We demonstrate that the IVD can be harvested and maintained as an organ unit with its native phenotype intact. We also demonstrate a high degree of genetic tractability in the model as we successfully recombined explants harvested from mice bearing conditional mutations (TGFßR1^fl/fl^, *mT/mG*; and *NF1^fl/fl^*). Given the recent advances in genetically-engineered mouse models [38], [39], the impact of genetic factors on IVDD progression can now be studied in greater detail.

We also demonstrate that *ex vivo* murine explants maintain their ability to respond to both catabolic and anabolic signals based on expression profiling of key structural and extracellular matrix genes. While there is upregulation of the aggrecanase *ADAMTS4* over time in culture, there appears to be a compensatory increase in the metalloproteinase inhibitor *TIMP1*, indicating that IVD explants retain a homeostatic drive under *ex vivo* conditions. In human tissue, aggrecanase expression is upregulated in degenerative IVDs [40]. The *murine* IVD may be a useful model to help elucidate the mechanistic role aggrecanases contribute to IVDD as they are candidate drug targets in other models of arthritis [41], [42]. It is worth noting that polymorphisms in the aggrecan gene are associated with the development of IVDD [43] and herniated discs are common in aggrecan^(+/−)^ mice [44].

Additionally, we induced a degenerative state under *ex vivo* culture conditions using exogenous IL-1ß, which is known to be upregulated in degenerative human IVDs [29], [45]. Our findings parallel those of others insofar as *murine* explants demonstrate downregulated aggrecan gene expression, upregulated *ADAMTS4* gene expression, and increased activated MAPK protein expression in response to IL-1ß stimulation [32]. Genetically engineered *murine* IVD explants may be a useful adjunct to characterize how the organ as a whole responds to inflammatory signals.

It is important to recognize current IVD explant models [9], [10], [11], [12], [13], [14], [15], [16], [17]. Because a strong genetic influence in IVDD exists [20], [21], [46], an *ex vivo* model of IVDD that allows efficient manipulation of both genetic and micro-environmental parameters will help broaden our understanding of targetable mechanisms underlying IVDD. As proof of concept, we assessed the murine IVD explant response to TGF-ß signaling. Exogenous TGF-ß3 treatment in rat explants resulted in upregulated expression of aggrecan and decreased degradation products [10]. Additionally, endogenous TGF-ß expression in disc tissue was shown to be protective by suppressing an inflammatory state [34] and upregulating connective tissue growth factor [35]. Our results with exogenous TGF-ß3 treatment confirm upregulated *ACAN* and *TIMP1* gene expression, consistent with previous studies in whole IVD explants [10]. Furthermore, by utilizing the *TGFßR1^fl/fl^* genetic construct, we also demonstrated significant upregulation of *ACAN* gene expression as a direct result of constitutive *TGF-ßR1* activation. These data confirm that by activating TGF-ß signaling at the receptor-level, either through direct administration of ligand or genetic recombination, we were able to increase gene expression of the principal NP proteoglycan, aggrecan.

It is important to recognize the limitations of our model and how other models may be better suited for certain investigations. While we can manipulate micro-environmental and genetic factors to recapitulate disc degeneration over 14 days, IVDD in humans is a chronic process spanning many years. Therefore, observations gleaned with the *murine* IVD are limited in this regard and would certainly benefit from human corollary data. Additionally, as the size and geometry of larger animal explant models more closely resemble the human IVD, biomechanical testing may be better suited for these models [11], [16]. Experimental surgical interventions are well studied in larger animal models and would be difficult to perform in the *murine* spine. Reproducible IVDD with needle puncture of the AF is well characterized in the rabbit spine [47] and *in vivo* biophysical parameter measurements, such as intradiscal pressure, have been measured in the *ovine* spine [48].

## Conclusions

The *murine* IVD explant is a useful tool to study the IVD. Under standard culture conditions the microanatomy of the IVD remains intact, the cells are viable, and the genetic expression pattern is consistent with an intact homeostatic drive. The *murine* IVD explant can be manipulated to induce a degenerative state or protective state. It can also be readily modified to reflect the diverse genetic factors underlying IVDD. We expect that this model will facilitate a more complete understanding of the complex mechanisms underlying IVDD.

## Supporting Information

Figure S1 (**a**) IVDs from wild-type (WT) and NF1^fl/fl^ mice were genotyped with allele specific PCR. (**b**) IVDs from wild-type (control) and NF1^fl/fl^ were cultured with cre-expressing adenovirus. Allele specific PCR demonstrates successful *ex vivo* genetic recombination in all IVDs tested.
(TIF)
