# Supplementary figures and images for: Genetic and Functional Studies of the Intervertebral Disc: A Novel Murine Intervertebral Disc Model
Source: PLoS One. 2014 Dec 4;9(12):e112454. doi: 10.1371/journal.pone.0112454 (PMC4256369; doi:10.1371/journal.pone.0112454)

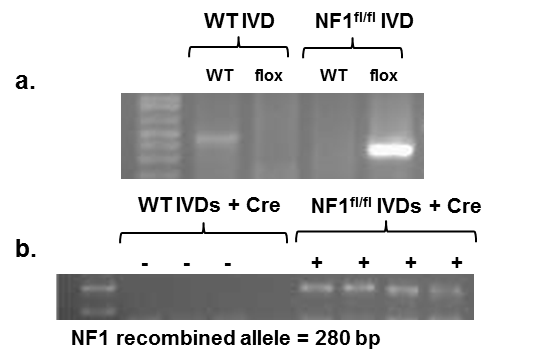

Supplement: Figure S1 — (a) IVDs from wild-type (WT) and NF1fl/fl mice were genotyped with allele specific PCR. (b) IVDs from wild-type (control) and NF1fl/fl were cultured with cre-expressing adenovirus. Allele specific PCR demonstrates successful ex vivo genetic recombination in all IVDs tested. (TIF) [file pone.0112454.s001.tif]
